# Fluocinolone acetonide intravitreal implant as a therapeutic option for severe Sjögren’s syndrome-related keratopathy: a case report

**DOI:** 10.1186/s13256-018-1916-4

**Published:** 2019-01-24

**Authors:** Joanna Wasielica-Poslednik, Norbert Pfeiffer, Adrian Gericke

**Affiliations:** Department of Ophthalmology, University Medical Center of the Johannes Gutenberg-University Mainz, Langenbeckstrasse 1, 55131 Mainz, Germany

**Keywords:** Secondary Sjögren’s syndrome, Fluocinolone acetonide intravitreal implant, Keratolysis, Penetrating keratoplasty

## Abstract

**Background:**

In this report, we present the results of a severe case of Sjögren’s syndrome-related keratopathy after fluocinolone acetonide 190-μg intravitreal implant (Iluvien®; Alimera Sciences Inc.) therapy.

**Case presentation:**

A 52-year-old Caucasian woman with Sjögren’s syndrome secondary to autoimmune hepatitis and primary sclerosing cholangitis was admitted to our emergency department owing to bilateral corneal ulcers and corneal perforation in the left eye following exposure keratopathy in an artificially induced coma. Within the following months, recurrent fulminant keratolysis with perforations required multiple penetrating keratoplasties and amniotic membrane transplants in both eyes. With new signs of severe keratolysis, an intravitreal fluocinolone acetonide implant was injected off-label in the left eye, and a third penetrating keratoplasty was performed 2 weeks later. In the 6 months of follow-up after the last penetrating keratoplasty, no more surgical interventions were needed in the eye with the fluocinolone acetonide implant. The corneal surface remained stable, and intraocular pressure was normal. During this time frame, two further penetrating keratoplasties, one vitrectomy, and five amniotic membrane transplants were performed in the fellow eye owing to relapsing keratolysis and perforations.

**Conclusions:**

To the best of our knowledge, this is the first report of fluocinolone acetonide intravitreal therapy in a patient with corneal disease. In the 6-month follow-up period, no surgical intervention was needed in the eye with the fluocinolone acetonide implant, whereas further penetrating keratoplasties and amniotic membrane transplants were performed in the fellow eye. Intravitreal fluocinolone acetonide may be considered as a treatment option in severe cases of autoimmune corneal disease.

## Background

Sjögren’s syndrome (SS) is a progressive autoimmune disease affecting exocrine glands and leading to oral and ocular dryness [1]. SS resulting from other inflammatory or rheumatic disorders is called secondary SS. Secondary SS is diagnosed in 7% of patients with autoimmune hepatitis (AIH) [2].

The treatment of SS-related dry eye disease includes topical and systemic drugs [3]. The most common and effective topical therapy options for dry eye disease are artificial tears, autologous serum, steroidal eye drops, cyclosporine, bandage contact lenses, and lacrimal punctal occlusion [4]. Systemic medications include secretagogues; metalloproteinase inhibitor doxycycline; and immunosuppressive drugs such as azathioprine, hydroxychloroquine, or prednisolone.

Fluocinolone acetonide (FAc) 190-μg intravitreal implant (Iluvien®; Alimera Sciences Inc., Alpharetta, GA, USA) has been approved for the treatment of persistent diabetic macular edema. The FAc implant provides stable long-term release of FAc with peak levels in the aqueous humor slightly above 2 ng/ml for approximately 3 months followed by steady-state levels between 1.0 and 0.5 ng/ml between 6 and 36 months [5]. In this report, we describe the first case where the FAc implant was used in a patient with severe SS-related keratopathy. The rationale was to provide the cornea with a constant intraocular level of corticosteroids because topical and systemic therapy seemed to be insufficient in preventing recurrent keratolysis.

## Case presentation

**Fig. 1:**
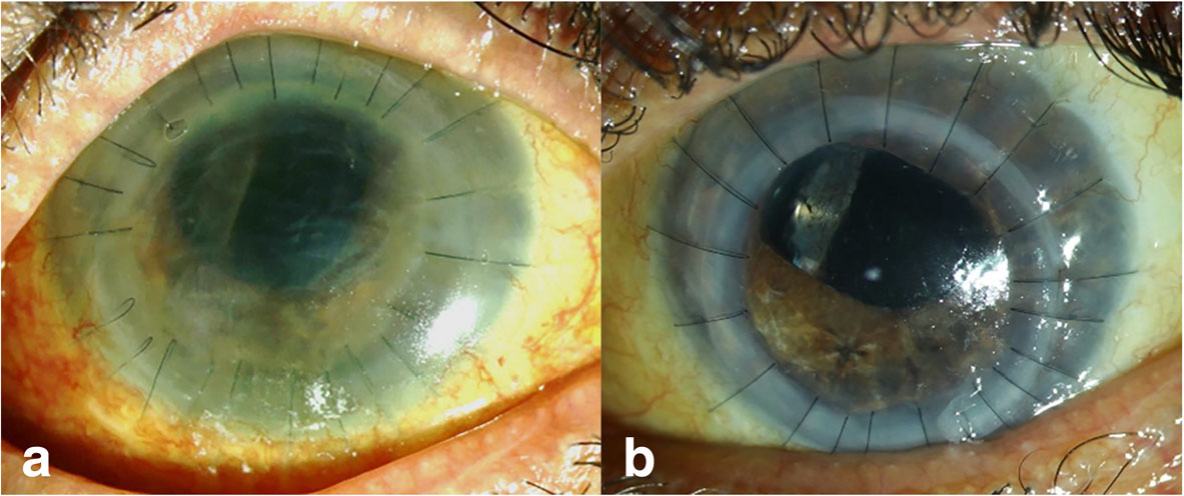
**a** The left eye at the time the fluocinolone acetonide implant was injected shows progressive corneal melting, Descemet folds, and loose sutures. **b** The left eye 7 months after fluocinolone acetonide injection and 6 months after the last penetrating keratoplasty

A 52-year-old Caucasian woman with SS secondary to AIH/primary sclerosing cholangitis overlap was admitted to our emergency department owing to bilateral keratolysis and corneal perforation in the left eye. The patient had had filiform keratitis and recurrent erosions for the previous 3 years. The dramatic worsening of her dry eye disease followed corneal exposure in an artificially induced coma during her stay on an intensive care unit owing to sigmoid colon perforation and sepsis. At the time of admission, the patient’s right eye had deep corneal melting, and best corrected visual acuity (BCVA) was 0.2 decimal. In the left eye, there was a corneal perforation, and BCVA was hand motions. Table 1 documents the surgical therapies performed in the right and left eyes owing to fulminant relapses of keratolysis and corneal perforations in the subsequent 10 months. Postoperative topical therapy consisted of dexamethasone disodium phosphate 1 mg/ml six times per day, cyclosporine 0.1% twice per day, ofloxacin eye drops four times per day, and hourly application of artificial tears and human albumin. Additionally, mycophenolate mofetil (2 g/day) was administered systemically. An enhancement of the systemic immunosuppression by corticosteroids or azathioprine was contraindicated because the patient had a history of sepsis [6]. Intravitreal injection of the FAc implant was performed off-label in her left eye 2 weeks after the second penetrating keratoplasty (PKP) because of new signs of corneal melting (Fig. 1a) and was followed by the third PKP and amniotic membrane transplant (AMT) 2 weeks later.

**Table 1:** History of surgical therapies in the right and left eyes

2017	Right eye	Left eye
February (admission)	AMT	1. PKP + ECCE + AMT
April	1. PKP + ECCE + AMT	AMT
May	2. PKP + AMT	2. PKP + AMT Iluvien injection (+ 2 weeks)
June		3. PKP + AMT+ tarsorrhaphy
July	AMT	
August	AMT 3. PKP + AMT + tarsorrhaphy	
September	4. PKP + PPV + AMT + tarsorrhaphy	
November	AMT + tarsorrhaphy	

*Abbreviations: AMT* Amniotic membrane transplant, *PKP* Penetrating keratoplasty, *PPV* Pars plana vitrectomy

In the 6 months of follow-up after the third PKP, no more surgical interventions were needed in the left eye that had been treated with the FAc implant. In this eye, there was a closed epithelium, BCVA was 0.16, intraocular pressure was normal without any intraocular pressure-lowering medication (Fig. 1b). However, during this period, two further PKPs, one vitrectomy, five AMTs, and three tarsorrhaphies were performed in the right eye owing to recurrent keratolysis and perforations (Table 1).

## Discussion

Dry eye disease associated with SS may have dramatic consequences for the integrity of the eye. Our patient had eight corneal perforations (five in the right eye and three in the left eye) within a 9-month period. Despite intensive ophthalmological care in a tertiary referral hospital and exhaustive surgical and medical therapy, new breakdowns of the corneal wound healing leading to fulminant keratolysis and perforations recurred. Because of new signs of corneal melting, a decision was made to treat one eye with an intravitreal FAc implant. Topical and systemic therapies were continued throughout and equally affected both eyes.

Although the concentration of the corticosteroid in the aqueous humor following intravitreal administration seems to be lower than topical applications, the low and sustained level may work to complement corticosteroids administered topically and systemically [6, 7]. A further advantage of administering an intravitreal drug is the low risk of systemic side effects and that it also does not stress the vulnerable corneal surface.

There was no need for any surgical intervention in the 6 months of follow-up in the eye with the FAc implant. Despite identical topical and systemic therapies being administered in both eyes and an almost identical clinical situation at the start of therapy, the fellow eye not treated with the FAc implant required further surgeries.

To the best of our knowledge, this is the first case where an intravitreal corticosteroid implant has been used to treat severe SS-related keratopathy. The outcome of the eye with the FAc implant was markedly better than that of the fellow eye in the 6-month follow-up. The intravitreal FAc implant may potentially supplement exhaustive topical and systemic immunosuppressive therapies used in severe cases of autoimmune corneal diseases and even in recurrent corneal graft rejections.

## Abbreviations

AIH: Autoimmune hepatitis
AMT: Amniotic membrane transplant
BCVA: Best corrected visual acuity
FAc: Fluocinolone acetonide
PKP: Penetrating keratoplasties
SS: Sjögren’s syndrome
